# LSTM-Enhanced TD3 and Behavior Cloning for UAV Trajectory Tracking Control

**DOI:** 10.3390/biomimetics10090591

**Published:** 2025-09-04

**Authors:** Yuanhang Qi, Jintao Hu, Fujie Wang, Gewen Huang

**Affiliations:** 1 School of Computer Science, University of Electronic Science and Technology of China, Zhongshan Institute, Zhongshan 528402, China; qiyuanhang@zsc.edu.cn; 2 College of Excellent Engineers, Dongguan University of Technology, Dongguan 523820, China; 221115202@dgut.edu.cn; 3 Modern Educational Technology Center, Jiaying University, Meizhou 514015, China; hgw@jyu.edu.cn

**Keywords:** deep reinforcement learning, UAV control, TD3 algorithm, LSTM, behavior cloning, target tracking

## Abstract

Unmanned aerial vehicles (UAVs) often face significant challenges in trajectory tracking within complex dynamic environments, where uncertainties, external disturbances, and nonlinear dynamics hinder accurate and stable control. To address this issue, a bio-inspired deep reinforcement learning (DRL) algorithm is proposed, integrating behavior cloning (BC) and long short-term memory (LSTM) networks. This method can achieve autonomous learning of high-precision control policy without establishing an accurate system dynamics model. Motivated by the memory and prediction functions of biological neural systems, an LSTM module is embedded into the policy network of the Twin Delayed Deep Deterministic Policy Gradient (TD3) algorithm. This structure captures temporal state patterns more effectively, enhancing adaptability to trajectory variations and resilience to delays or disturbances. Compared to memoryless networks, the LSTM-based design better replicates biological time-series processing, improving tracking stability and accuracy. In addition, behavior cloning is employed to pre-train the DRL policy using expert demonstrations, mimicking the way animals learn from observation. This biomimetic plausible initialization accelerates convergence by reducing inefficient early-stage exploration. By combining offline imitation with online learning, the TD3-LSTM-BC framework balances expert guidance and adaptive optimization, analogous to innate and experience-based learning in nature. Simulation experimental results confirm the superior robustness and tracking accuracy of the proposed method, demonstrating its potential as a control solution for autonomous UAVs.

## 1. Introduction

With the development of technology, UAVs have been increasingly used in disaster relief, agricultural plant protection, logistics and distribution, wind farm operation and maintenance, and other fields [1]. Inspired by biomimetic principles, recent studies have sought to enhance UAV adaptability and robustness by mimicking biological learning and control mechanisms. Classic control algorithms such as PID control [2], model predictive control (MPC) [3], and sliding mode control [4] rely on accurate system dynamics modeling and perform well in structured environments. In order to enhance the adaptability under dynamic conditions such as load changes and wind disturbances, an adaptive PID strategy is proposed in [5] to improve flight stability by adjusting control parameters in real time. In [6], a PID-based pitch control system is developed for UAV trajectory tracking near or above the speed of sound. In [7], a stable adaptive PID control scheme for UAV systems is introduced. It aims to accurately estimate the ideal controller to satisfy a particular control objective and dynamically adjust its gains through a stable adaptive process. Aiming at the problem that the deception performance is greatly reduced due to the sudden change of UAV trajectory, a UAV dynamic trajectory deception method based on MPC is proposed in [8], which gradually guides the UAV towards the predetermined trajectory of the deceiver. Aiming at the target trajectory planning problem of UAV swarm in uncertain environments, an optimized MPC method based on a deep neural network (DNN) is proposed in [9]. A UAV trajectory planning algorithm based on a nonlinear MPC scheme is studied in [10]. By controlling the parameters, the UAV trajectory planning problem is transformed into a nonlinear planning problem, thereby alleviating the heavy computational burden of solving the NMPC optimization problem online. In [11], a time-delay sliding mode is proposed to resolve the control problem of the manipulator. The sliding surface is composed of the sliding variable value at the previous sampling moment, which enhances the tracking performance with fast convergence speed and minimum steady-state error. In [12], it proposes an adaptive fuzzy fixed-time sliding mode formation control method for quadrotor UAVs, which can achieve the predetermined performance under uncertainty and external interference. Although traditional control methods are widely used in UAV control, these methods often require complex parameter tuning and online optimization, and their adaptability is limited. In [13], a safety control method based on event triggering and adaptive dynamic programming was proposed to solve the control problem of nonlinear systems with asymmetric input constraints and state constraints. For unknown nonlinear systems with actuator failures and asymmetric input constraints, a safe and optimal fault-tolerant control method based on a control barrier function and neural network was proposed in [14], and its effectiveness was verified through theoretical analysis and simulation.

In order to break through the bottleneck of traditional methods, reinforcement learning (RL) has shown significant advantages by learning the optimal strategy through the trial and error mechanism and interaction with the environment. In [15], it proposes a deep reinforcement learning UAV navigation method, which uses adaptive control and an attention mechanism to dynamically balance navigation and obstacle avoidance and optimizes control performance through speed constraint loss. In [16], a fixed-wing UAV trajectory tracking control method based on deep deterministic policy gradient (DDPG) is proposed. In [17], a Z-function decomposition-based RL approach has been developed to jointly optimize transmission power and UAV trajectories, aiming to improve target positioning accuracy. To improve the robustness of the nonlinear dynamic inversion (NDI) method under model uncertainty, a control scheme combining a TD3 agent with the NDI method is proposed in [18]. In [19], a hierarchical PPO reinforcement learning method is proposed to improve the maneuverability of UAVs in complex environments by decomposing high-level and low-level tasks. In [20], a reinforcement learning (RL)-based quadrotor control architecture is proposed, using the Soft Actor-Critic algorithm, a model-free off-policy stochastic RL algorithm, to train the RL agent. However, standard DRL algorithms still face challenges in handling time-dependent tasks such as long-term trajectory prediction such as drone trajectory tracking.

Therefore, the researchers introduced an LSTM network to enhance the ability of the policy network to remember historical states [21]. In [22], the application of DRL in the trajectory tracking of a mobile robot with a sliding steering under terrain constraints is discussed, and it is proposed to integrate LSTM into the DRL controller to address partial observability issues in navigation. To address the problem of the poor obstacle avoidance effect of the particle model in an ideal environment, a non-particle model USV obstacle avoidance algorithm based on LSTM-PPO is proposed in [23], and a training environment that adapts to non-ideal environments is constructed. In order to solve the tracking control problem of the manipulator, a method based on LSTM and generative adversarial imitation learning (GAIL) is proposed in [24]. In [25], an improved deep reinforcement learning algorithm based on LSTM and MATD3 is proposed for training multi-UAV adaptive collaborative formation trajectory planning. In addition, imitation learning methods such as BC are also widely used to assist reinforcement learning training, especially when data samples are insufficient or the performance of the strategy is poor in the early stage of training. In [26], a BC and PPO based approach is proposed to solve the within visual range (WVR) air-to-air combat problem of aircraft and missiles under complex nonlinear six-degree-of-freedom (6-DOF) dynamics. In [27], a collaborative method combining reinforcement learning and imitation learning is proposed to solve the problem of ordinary reinforcement learning having a poor learning effect on navigation policy in partially observable non-Markov environments.

In summary, combining deep reinforcement learning with the temporal modeling structure and imitation learning mechanism has become an effective way to improve the intelligent control performance of UAVs [28]. This paper aims to build a deep reinforcement learning control framework that integrates LSTM structure and acbehavior cloning mechanism and applies it to the three-dimensional trajectory tracking task of UAVs with six-degree-of-freedom dynamic modeling. The main contributions are as follows:

(1) Inspired by references [22,23,24], an LSTM network is introduced to enhance the trajectory tracking capability of drones in dynamic environments. By mimicking the memory and temporal processing capabilities of biological neural systems, the LSTM layer extracts time-dependent features from the sequential observations of the UAV. These features are processed by the policy network to generate continuous control actions, which significantly improves the adaptability and control stability of the algorithm in partially observable environments.
(2) Based on the advantages of the BC method, this paper innovatively combines expert demonstration data with reinforcement learning by using the principle of biomimetics. By pre-training the policy network to imitate the expert behavior, it not only greatly shortens the exploration time in the early stage of training but also avoids the generation of dangerous control actions.
(3) A TD3-LSTM-BC algorithm that integrates TD3, LSTM, and BC is proposed for drone trajectory tracking control. The algorithm captures the time-series dependency through LSTM and uses BC to provide expert prior knowledge, achieving the coordinated optimization of control accuracy, learning efficiency, and robustness under the TD3 framework.

## 2. Unmanned Aerial Vehicle System Modeling

The UAV is modeled as a 6-degree-of-freedom (6-DoF) rigid body with states including position, velocity, Euler angles, and angular velocities. The dynamics are derived using Newton–Euler equations, considering forces and moments acting on the UAV [29]. The position $P={\left[p_{x},p_{y},p_{z}\right]}^{T}$ and velocity $V={\left[v_{x},v_{y},v_{z}\right]}^{T}$ evolve according to:

(1) $$\begin{array}{c} \dot{p}=v\\ \dot{v}=\frac{F}{m}+g \end{array}$$

where *m* is the UAV mass $F={\left[F_{x},F_{y},F_{z}\right]}^{T}$ is the applied force (control input), and $g={\left[0,0,-9\right]}^{T}$ is the gravity. In this implementation, the control input is applied directly as linear acceleration:

(2) $$\begin{array}{c} v_{t+1}=v_{t}+a_{linear}\cdot \Delta t\\ p_{t+1}=p_{t}+v_{t}\cdot \Delta t \end{array}$$

where $a_{linear}=F/m$ is the commanded linear acceleration.

The attitude is represented in Euler angles $\Theta ={\left[\phi ,\theta ,\psi \right]}^{T}$, and the angular velocity is $\omega ={\left[\omega_{x},\omega_{y},\omega_{z}\right]}^{T}$. The dynamics are given by:

(3) Θ=R(Θ).ω

where R(Θ) is the transformation matrix from body rates to Euler angle derivatives. The angular acceleration is controlled directly:

(4) $$\begin{array}{c} \omega_{t+1}=\omega_{t}+a_{linear}\cdot \Delta t\\ \Theta_{t+1}=\Theta_{t}+\omega_{t}\cdot \Delta t \end{array}$$

where $a_{linear}$ is the commanded angular acceleration. To prevent unrealistic attitudes, Euler angles are constrained:

(5) $$\phi ,\theta \in \left[-\frac{\pi }{2},\frac{\pi }{2}\right],\;\psi \in \left[-\pi ,\pi \right]$$

## 3. Preliminaries

### 3.1. Reinforcement Learning

RL is a learning paradigm where an agent interacts with an environment modeled as a Markov Decision Process (MDP) defined by the tuple (S,A,P,r,γ) [30], where:

– S is the state space,
– A is the action space,
– $P\left(s_{t+1}|s_{t},a_{t}\right)$ is the transition probability,
– $r\left(s_{t},a_{t}\right)$ is the reward function,
– γ∈[0,1] is the discount factor.

The agent aims to learn a policy $\pi (a_{t}|s_{t})$ that maximizes the expected discounted return:

(6) $$J\left(\pi \right)=E_{\pi }\left[\sum_{t=0}^{\infty }\gamma^{t}r\left(s_{t},a_{t}\right)\right]$$

In continuous control tasks like UAV trajectory tracking, actor-critic methods are widely used, where:

– the actor $\pi_{\theta }\left(a|s\right)$ outputs continuous actions,
– the critic $Q_{\phi }\left(s,a\right)$ estimates the action-value function:

(7) $$Q^{\pi }\left(s_{t},a_{t}\right)=E_{\pi }\left[\sum_{t^{\prime }=t}^{\infty }\gamma^{t^{\prime }-t}r\left(s_{t^{\prime }},a_{t^{\prime }}\right)\right]$$

### 3.2. TD3 Algorithm

TD3 is an advanced off-policy algorithm that is mainly used in continuous action spaces. It addresses the overestimation bias of Q-values found in DDPG by introducing three key techniques:

1. Clipped Double Q-learning: maintain two critics $Q_{\phi_{1}}$ and $Q_{\phi_{2}}$, and use the smaller Q-value in the target:
  (8) $$y_{t}=r_{t}+\gamma \cdot \min_{i=1,2}Q_{\phi_{i}^{\prime }}\left(s_{t+1},\pi_{\theta^{\prime }}\left(s_{t+1}\right)+\epsilon \right)$$
  where ϵ∼clip(N(0,σ),−c,c) adds noise for target smoothing.
2. Delayed Policy Update: to reduce the discrepancy, the actor network is updated less frequently than the critic network.
3. Target Policy Smoothing: adds noise to the target action for smoother Q-function estimation.

The critic loss is defined as:

(9) $$L_{Q}=E_{\left(s_{t},a_{t},r_{t},s_{t+1}\right)∼D}\left[{\left(Q_{\phi_{i}}\left(s_{t},a_{t}\right)-y_{t}\right)}^{2}\right]$$

The actor is updated to maximize the critic’s estimate:

(10) $$L_{\pi }=-E_{s_{t}∼D}\left[Q_{\phi_{1}}\left(s_{t},\pi_{\theta }\left(s_{t}\right)\right)\right]$$

### 3.3. Long Short-Term Memory

To capture temporal dependencies and history in UAV control tasks, this paper embeds an LSTM network in the policy and/or critic. LSTM is a type of recurrent neural network (RNN) designed to alleviate the vanishing gradient problem through a gating mechanism [31]. At each time step *t*, given input $x_{t}\in R^{d}$, previous hidden state $h_{t-1}$, and cell state $C_{t-1}$, the LSTM performs the following updates:

$$\begin{array}{cccc} f_{t} & =\sigma \left(W_{f}\left[h_{t-1},x_{t}\right]+b_{f}\right) & & \;(\mathrm{forget}\;\mathrm{gate})\;\\ i_{t} & =\sigma \left(W_{i}\left[h_{t-1},x_{t}\right]+b_{i}\right) & & \;(\mathrm{input}\;\mathrm{gate})\;\\ {\tilde{C}}_{t} & =\tanh\left(W_{C}\left[h_{t-1},x_{t}\right]+b_{C}\right) & & \;(\mathrm{candidate}\;\mathrm{cell}\;\mathrm{state})\;\\ C_{t} & =f_{t}⊙C_{t-1}+i_{t}⊙{\tilde{C}}_{t} & & \;(\mathrm{cell}\;\mathrm{state}\;\mathrm{update})\;\\ o_{t} & =\sigma \left(W_{o}\left[h_{t-1},x_{t}\right]+b_{o}\right) & & \;(\mathrm{output}\;\mathrm{gate})\;\\ h_{t} & =o_{t}⊙\tanh\left(C_{t}\right) & & \;(\mathrm{new}\;\mathrm{hidden}\;\mathrm{state})\; \end{array}$$

where:

– σ(·) denotes the sigmoid activation,
– ⊙ denotes element-wise multiplication,
– $W_{f},W_{i},W_{C},W_{o}$ and $b_{f},b_{i},b_{C},b_{o}$ are weight matrices and bias vectors.

The LSTM is integrated into the policy and/or value networks to process sequences of states and actions, which is beneficial in partially observable or history-dependent UAV environments [32].

### 3.4. Behavior Cloning

Behavior cloning is a form of supervised imitation learning that learns a policy from expert demonstrations. Given an expert dataset:

$$D_{E}={\left\{\left(s_{i},a_{i}^{*}\right)\right\}}_{i=1}^{N}$$

where:

– $s_{i}\in S$ is the observed state,
– $a_{i}^{*}\in A$ is the expert’s action.

The policy $\pi_{\theta }\left(a|s\right)$ is trained to minimize the mean squared error (or other divergence metrics) between its output and the expert action:

$$L_{BC}\left(\theta \right)=\frac{1}{N}\sum_{i=1}^{N}{∥\pi_{\theta }\left(s_{i}\right)-a_{i}^{*}∥}^{2}$$

This provides a warm start for reinforcement learning, accelerating convergence and improving safety during early exploration. BC can also be integrated into TD 3, resulting in TD3-BC, by regularizing the policy loss:

$$L_{\pi }=\lambda \cdot L_{BC}-\left(1-\lambda \right)\cdot E_{s∼D}\left[Q_{\phi }\left(s,\pi_{\theta }\left(s\right)\right)\right]$$

where:

– λ∈[0,1] is a weighting coefficient balancing between imitation and value maximization,
– $Q_{\phi }$ is the critic network.

This hybrid objective ensures that the policy remains close to expert behavior while still exploring higher-reward actions via RL optimization [33].

## 4. Control Design

### 4.1. Input and Output

The state space is designed to comprehensively capture the UAV’s full motion state and tracking objectives, including position, velocity, orientation (Euler angles), angular velocity, and the target position. This allows the learning algorithm to observe both the current dynamic status and the goal, enabling more accurate and responsive control decisions. The full state vector is:

(11) $$s_{t}=\left[p,v,\Theta ,\omega ,p_{target}\right]\in R^{15}$$

where $p_{target}$ is the current target position.

The action space, defined as linear and angular accelerations, provides direct and interpretable control over both translational and rotational dynamics. This setup strikes a balance between model complexity and learning efficiency, avoids unnecessary coupling from low-level motor dynamics, and facilitates smooth policy learning in continuous control tasks like trajectory tracking. The agent outputs a six-dimensional continuous action:

(12) $$a_{t}=\left[a_{linear},a_{angular}\right]\in R^{6}$$

To guide the UAV toward accurate trajectory tracking, the reward function is designed based on the position error $e_{t}$, defined as:

(13) $$e_{t}=p-p_{target}$$

A smoothed negative L1-norm reward is used to ensure continuous gradients and robustness:

(14) $$r_{t}=-\lambda ||e_{t}{\left|\right|}_{1}=-\frac{1}{3}\lambda \sum_{i=1}^{3}\left|p_{i}-p_{target\;,i}\right|$$

where λ is a weight coefficient between (0, 1). This formulation penalizes large deviations from the target and encourages the agent to minimize trajectory tracking error. The main goal of this task is to minimize the position error, and the reward function is essentially a linear penalty on the error, so the role of λ is only to normalize and scale the reward value without changing the training objective or the optimal policy.

**Remark** **1.** *In real UAV control tasks, the environmental state has time dependence. Traditional MLP strategies have difficulty capturing this temporal feature, resulting in poor policy generalization. The LSTM module can explicitly model the temporal relationship between state sequences, making the strategy more adaptable in partially observable and dynamic environments, thereby improving control accuracy and stability [24].*

### 4.2. TD3-LSTM Design

Most reinforcement learning algorithms use the actor-critic architecture to efficiently deal with policy gradient problems. In this architecture, the policy network (Actor) is continuously optimized under the evaluation feedback of the value network (Critic) and finally learns a policy π for performing trajectory tracking control tasks. The core task of the Critic network is to evaluate the performance of the current policy and provide learning signals for policy updates.

The TD3-LSTM algorithm designed in this paper is based on the actor-critic framework and is mainly composed of a policy network and a value network. For the trajectory tracking task, the state of the UAV agent at each time step constitutes a state sequence and is input to the LSTM network. The LSTM generates the corresponding hidden state *h* at each time step and uses it as the input of the subsequent fully connected layer. After being processed by the fully connected network, the policy network outputs the Gaussian distribution parameters of the action at that time step. During the training process, actions are sampled from this distribution to enhance the randomness and exploration ability of the policy.

The input of the value network is the concatenation of the state sequence and the corresponding action. After passing through a fully connected network and an output layer, it outputs a scalar representing the state value $V_{\phi }\left(S\right)$ under the current state sequence, which is used to guide the update of the policy network.

### 4.3. TD3-LSTM-BC Design

On the basis of TD3-LSTM, a BC mechanism is further introduced to pre-train the policy network to improve the sample efficiency and policy stability in the early stage of learning, as shown in Algorithm 1. In this paper, the expert data primarily comes from trajectory data trained using the TD3 and TD3-LSTM algorithms. These strategies have demonstrated good stability and control performance in UAV trajectory tracking tasks and can therefore serve as reliable expert demonstrations. In the BC stage, the expert demonstration data $D^{*}=\left(s_{t-k:t}^{\left(j\right)},at^{*(j)}\right){j=1}^{N}$ is used to train the LSTM-Actor approximate expert policy with the state sequence as input.

Specifically, the state sequence with a time window length of *k* is first input into the LSTM network to extract the hidden state $h_{t}$, and then the policy network outputs the action $\pi \theta (h_{t})$. The training goal is to minimize the mean square error loss between the policy output and the expert action:

(15) $$L_{BC}\left(\theta \right)=\frac{1}{N}\sum_{j=1}^{N}{∥\pi_{\theta }\left(h_{t}^{\left(j\right)}\right)-a_{t}^{*(j)}∥}^{2}$$

Update the policy network parameters θ by gradient descent:

(16) $$\theta \leftarrow \theta -\alpha_{BC}\nabla_{\theta }L_{BC}$$

After completing several rounds of BC pre-training, the algorithm enters the online TD3 reinforcement learning phase and continues to optimize the Actor and Critic networks based on the environment interaction data. BC training only acts on the policy network, and the Critic network is still updated from the environment sampling to keep the modules decoupled.

**Remark** **2.** *In deep reinforcement learning, the initial strategy is often random, which can easily lead to inefficient exploration or state collapse in the early stages of training, especially in complex control tasks such as UAVs. By introducing expert data for behavior cloning, the strategy can be quickly guided to a reasonable strategy space, improving the initial performance of the strategy and reducing training interference caused by invalid actions. Combined with the time-dependent features extracted by LSTM, BC can significantly accelerate the convergence of the strategy while improving training stability and final performance.*

**Algorithm 1** TD3 with LSTM-Actor and BC for UAV Trajectory Tracking
  1:
**Initialize:**
  2:   LSTM-based policy network $\pi_{\theta }$, MLP critics $Q_{\psi_{1}},Q_{\psi_{2}}$  3:   Target networks $\pi_{\theta^{-}},Q_{\psi_{1}^{-}},Q_{\psi_{2}^{-}}\leftarrow$ copies of $\pi_{\theta },Q_{\psi_{1}},Q_{\psi_{2}}$  4:   Replay buffer D←∅, Expert demonstration buffer $D^{*}$  5:   Time window size *k*, batch size *B*, soft update rate τ  6:
**Phase 1: Behavior Cloning Pre-Training**
  7:**for** epoch=1 to $N_{BC}$ **do**  8:    Sample batch ${\left\{\left(s_{t-k:t}^{\left(j\right)},a_{t}^{*(j)}\right)\right\}}_{j=1}^{B}∼D^{*}$  9:    **for** j=1 to *B* **do**10:        Compute LSTM hidden state: $h_{t}^{\left(j\right)}\leftarrow {\mathrm{LSTM}}_{\theta }\left(s_{t-k:t}^{\left(j\right)}\right)$11:    **end for**12:    Compute BC loss: $L_{BC}=\frac{1}{B}\sum_{j=1}^{B}{∥\pi_{\theta }\left(h_{t}^{\left(j\right)}\right)-a_{t}^{*(j)}∥}^{2}$13:    Update θ using $\nabla_{\theta }L_{BC}$14:
**end for**
15:
**Phase 2: Online TD3 Training**
16:**for** episode=1 to *M* **do**17:    Initialize time series buffer with *k* frames: $s_{1:k}\leftarrow \mathrm{env}.\mathrm{reset}()$18:    **while** not done **do**19:        $h_{t}\leftarrow {\mathrm{LSTM}}_{\theta }\left(s_{t-k:t}\right)$20:        Select action with exploration noise: $a_{t}\leftarrow \pi_{\theta }\left(h_{t}\right)+N\left(0,\sigma \right)$21:        Execute $a_{t}$, observe $r_{t},s_{t+1},d_{t}$22:        Store transition $\left(s_{t-k:t},a_{t},r_{t},s_{t-k+1:t+1},d_{t}\right)$ in buffer D23:    **end while**24:    **if** |D|≥B **then**25:        **for** each gradient update step **do**26:           Sample mini-batch ${\left\{\left(s_{t-k:t},a_{t},r_{t},s_{t-k+1:t+1},d_{t}\right)\right\}}_{j=1}^{B}∼D$27:           Compute $h_{t}^{\left(j\right)}\leftarrow {\mathrm{LSTM}}_{\theta }\left(s_{t-k:t}^{\left(j\right)}\right)$28:           Compute $h_{t+1}^{\left(j\right)}\leftarrow {\mathrm{LSTM}}_{\theta^{-}}\left(s_{t-k+1:t+1}^{\left(j\right)}\right)$29:           Compute target action: $a_{t+1}^{\prime }\leftarrow \pi_{\theta^{-}}\left(h_{t+1}^{\left(j\right)}\right)+\mathrm{clip}\left(N\left(0,\tilde{\sigma }\right),-c,c\right)$30:           Compute target Q-value:$$y_{j}=r_{t}+\gamma \left(1-d_{t}\right)\cdot \min_{i=1,2}Q_{\psi_{i}^{-}}\left(h_{t+1}^{\left(j\right)},a_{t+1}^{\prime }\right)$$31:           Update critics by minimizing:$$L_{\psi_{i}}=\frac{1}{B}\sum_{j=1}^{B}{\left(Q_{\psi_{i}}\left(h_{t}^{\left(j\right)},a_{t}^{\left(j\right)}\right)-y_{j}\right)}^{2}$$32:        **end for**33:        **if** episode % 2 == 0 **then**34:           Update actor by policy gradient:$$\nabla_{\theta }J\approx \frac{1}{B}\sum_{j=1}^{B}\nabla_{\theta }Q_{\psi_{1}}\left(h_{t}^{\left(j\right)},\pi_{\theta }\left(h_{t}^{\left(j\right)}\right)\right)$$35:           Soft update targets:$$\psi_{i}^{-}\leftarrow \tau \psi_{i}+\left(1-\tau \right)\psi_{i}^{-},\;\theta^{-}\leftarrow \tau \theta +\left(1-\tau \right)\theta^{-}$$36:        **end if**37:    **end if**38:
**end for**



## 5. Simulation

In order to verify the effectiveness of the proposed TD3-LSTM-BC algorithm in the UAV trajectory tracking task, this chapter conducts comparative experiments based on the UAV simulation environment built in Section 2. All simulations of the UAV trajectory tracking task were implemented in Python 3.9 using the PyTorch 2.1.0 framework. The training process was conducted on a workstation equipped with an Intel i5-12500K CPU, 64 GB RAM, and a single NVIDIA RTX 2060S GPU. The total number of steps in the experiment is set to N=200, the step length is Δt=0.1 s, and the total simulation time of the system is T=N·Δt=20 s. Four benchmark algorithms, DDPG, TD3, TD3-LSTM, and TD3-BC, are selected for comparison. In this paper, a spiral trajectory is selected as the target trajectory, as shown below:

$${Targt}_{Traj}=\left[\begin{array}{c} x\\ y\\ z \end{array}\right]=\left[\begin{array}{c} 3\cos(0.3*t)\\ 3\sin(0.3*t)\\ 0.3*t \end{array}\right]$$

The trajectory defines a spiral motion with a constant radius, where the *x*- and *y*-axes form a circular motion with an angular frequency of ω=0.3 rad/s, and the *z*-axis climbs at a constant rate of 0.3 m/s. Compared with conventional spiral trajectories, this design enhances the challenge of trajectory tracking through time-varying parameters and can fully test the algorithm’s adaptability to nonlinear dynamic systems [34].

The experiment will conduct a quantitative analysis from three dimensions: the algorithm’s training effect, the controller’s trajectory tracking accuracy, and the speed accuracy and robustness. The parameters of the UAV are shown in Table 1.

### 5.1. Training Performance

**Figure 1:**
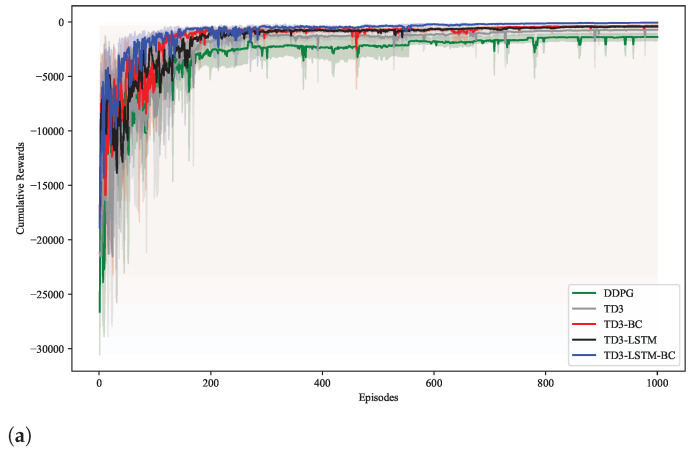

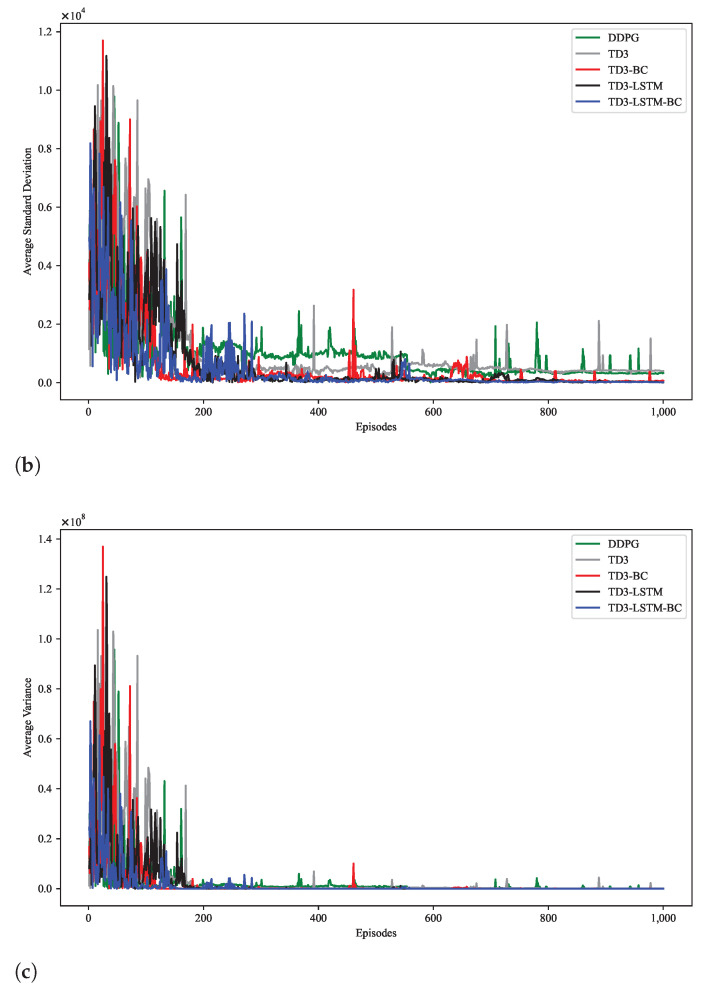
(**a**) Cumulative reward. (**b**) Standard deviation of cumulative reward. (**c**) Variance of cumulative reward.

In order to evaluate the training performance of each algorithm, all methods were trained for 1000 rounds in the same environment, and the experiment is repeated four times to eliminate the influence of randomness [35]. As shown in Figure 1, the reward curves of the five algorithms show obvious differences: The DDPG algorithm performs the worst among all methods, with the slowest convergence speed and the highest reward fluctuations throughout training. It frequently falls into local optima, failing to adapt effectively to the time-varying trajectory tasks due to its inherent limitations in policy updates and exploration–exploitation balance.

The traditional TD3 algorithm, while outperforming DDPG, still exhibits slower convergence compared to other variants, and its rewards show significant fluctuations even after stabilization, indicating insufficient policy stability in dynamic tasks. In contrast, the TD3-LSTM algorithm, with its LSTM structure, significantly improves training stability. Its reward curve variance is markedly lower than both TD3 and DDPG, demonstrating the effectiveness of LSTM in capturing temporal dependencies and enhancing policy robustness. The TD3-BC algorithm, combined with BC, achieves faster initial convergence by leveraging expert data to initialize the policy, thereby reducing inefficient exploration. However, its long-term stability remains inferior to TD3-LSTM-BC.

The TD3-LSTM-BC algorithm proposed in this paper synthesizes the strengths of both approaches. It converges faster than all baseline algorithms while maintaining the lowest reward fluctuations. This confirms that the prior knowledge of BC and temporal modeling capability of LSTM forms a synergistic effect, jointly optimizing policy convergence and stability in complex time-varying environments.

### 5.2. Tracking Control Performance

**Figure 2:**
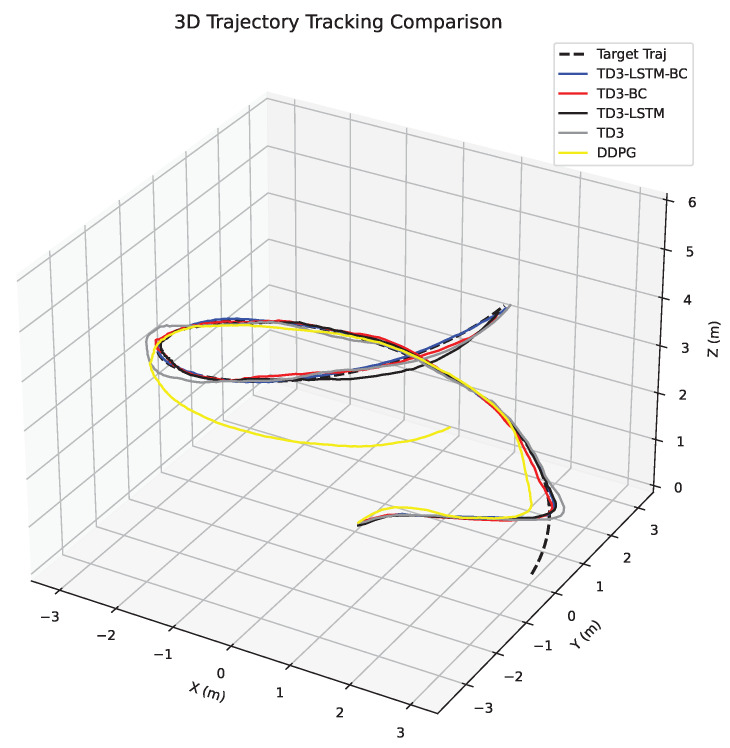
3D display of UAV tracking trajectory.

In order to evaluate the control performance of each algorithm, this section conducts a comparative analysis from two dimensions: trajectory tracking accuracy and speed error. Figure 2 shows the position tracking curves of five algorithms in three-dimensional space. The DDPG algorithm exhibits the poorest tracking performance, with severe deviations in all three axes, especially during turns and altitude changes. Its trajectory lags significantly behind the target and fails to recover, accumulating the largest final position error due to unstable policy updates and inadequate dynamic adaptation.

**Figure 3:**
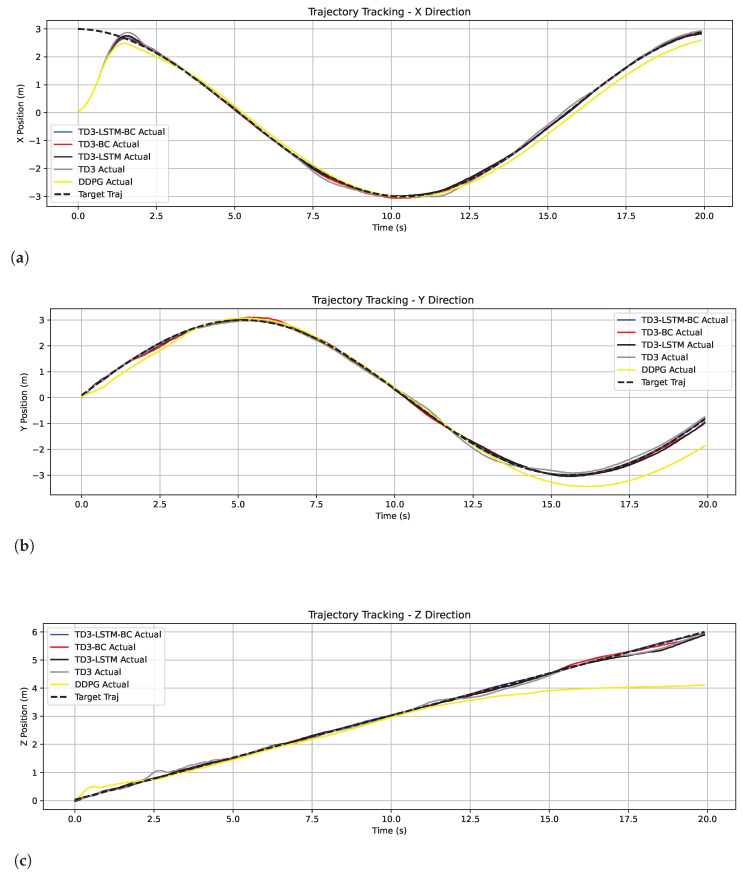
Tracking trajectories in the x, y, and z directions in Experiment 2. (**a**) Tracking trajectories in the x. (**b**) Tracking trajectories in the y. (**c**) Tracking trajectories in the z.

Among the remaining algorithms, TD3-LSTM-BC achieves the highest consistency with the target trajectory, demonstrating superior tracking precision. In contrast, TD3 shows obvious tracking lag during turns and altitude transitions, where its cumulative error is maximal. The tracking effects of TD3-LSTM and TD3-BC are comparable, both outperforming TD3 but slightly inferior to TD3-LSTM-BC. This confirms that while the LSTM structure enhances long-term dependency modeling and BC accelerates initial convergence, their isolated use still leaves room for improvement. Figure 3 shows the position tracking in the *x*, *y*, and *z* directions.

**Figure 4:**
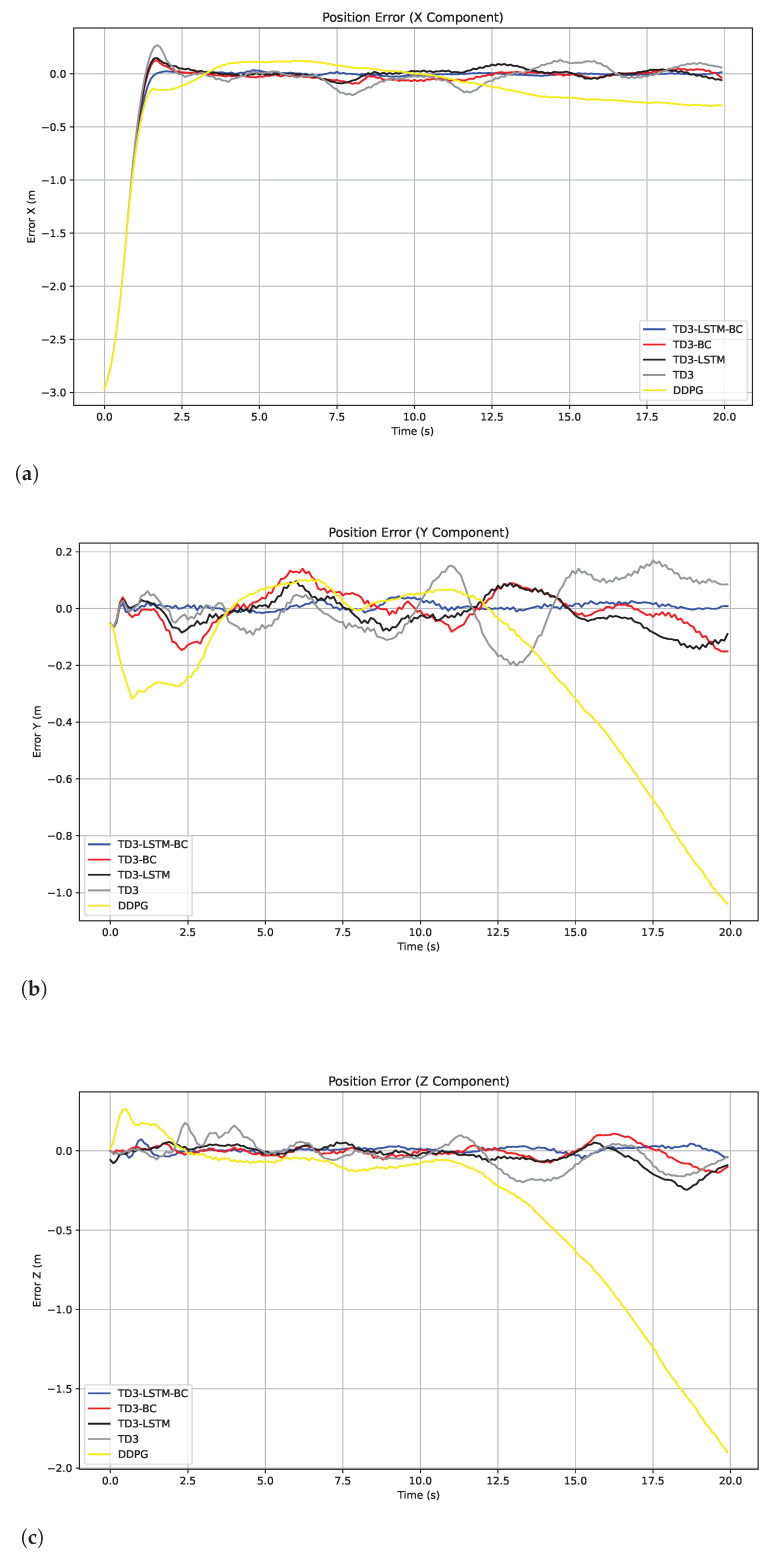
Tracking errors in the x, y, and z directions in Experiment 2. (**a**) Tracking errors in the x. (**b**) Tracking errors in the y. (**c**) Tracking errors in the z.

Further observation of the tracking error curve in Figure 4 reveals that TD3-LSTM-BC achieves significantly lower root mean square error (RMSE) and mean absolute error (MAE) compared to both the baseline TD3 and DDPG algorithms while maintaining the most stable error fluctuations across all three axes. DDPG exhibits the most unstable error profile, with its *y*-axis and *z*-axis error showing progressive divergence due to the compounding effects of spiral motion. While TD3 performs better than DDPG, it still demonstrates substantial oscillations, particularly in the *y*-axis direction. Both TD3-LSTM and TD3-BC show noticeable improvements over TD3, with TD3-LSTM displaying superior transient response in the *x*-axis and TD3-BC achieving better initial convergence—findings that align with the training performance analysis. The MAE and RMSE of the tracking position errors of the five algorithms are shown in Table 2.

**Figure 5:**
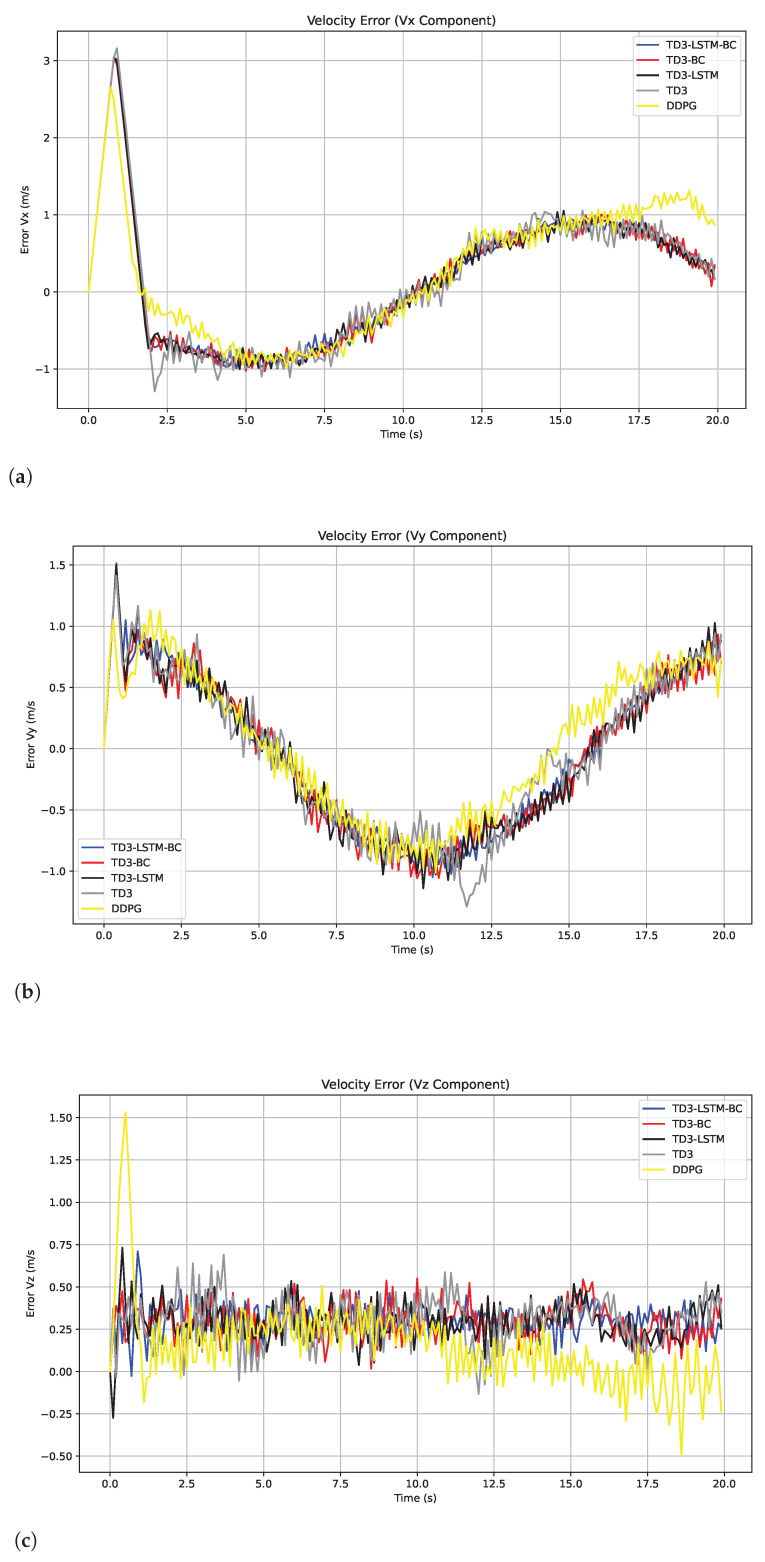
Tracking error of velocity in x, y, and z directions in Experiment 2. (**a**) Tracking error of velocity in x. (**b**) Tracking error of velocity in y. (**c**) Tracking error of velocity in z.

For speed tracking performance, TD3-LSTM-BC maintains its advantage, achieving consistently lower speed errors than all algorithms across dynamic maneuvers, as shown in Figure 5. Although DDPG shows a smaller steady-state speed error, this may be because its strategy is more likely to fall into the local optimum and learn a conservative “slow approach” control method. Although this strategy maintains a small speed difference, it leads to a large trajectory position error accumulation due to slow response and limited action range. TD3 improves upon the instability of DDPG but still suffers from speed overshoots in rapidly changing phases, as its deterministic policy lacks explicit memory of past states. In contrast, TD3-LSTM-BC effectively mitigates these issues by combining LSTM-based temporal modeling with BC-guided policy initialization, enabling smoother velocity tracking during complex, time-varying maneuvers. These results highlight that while the local optimum of DDPG is able to yield deceptively favorable speed errors in restricted scenarios, the integrated architecture of TD3-LSTM-BC delivers superior robustness and accuracy across the full trajectory spectrum. The MAE and RMSE of tracking velocity errors of five algorithms are shown in Table 3.

### 5.3. Anti-Disturbance Performance

In the simulation experiment, in order to more realistically simulate the actuator uncertainty and random disturbances that the UAV control system may encounter in the real environment, Gaussian action noise is introduced into the environment.

(17) $${\tilde{a}}_{t}=\mathrm{clip}\left(a_{t}+\varepsilon_{t},-a_{\max},a_{\max}\right),\;\varepsilon_{t}∼N\left(2,\sigma^{2}I_{d}\right)$$

Among them, ${\tilde{a}}_{t}$ denotes the final action executed in the environment after applying noise and clipping; $a_{t}$ is the original action output by the policy network; $\varepsilon_{t}$ is the additive Gaussian noise sampled from a two-mean multivariate normal distribution with covariance matrix $\sigma^{2}I_{d}$, where σ is the standard deviation of the noise and $I_{d}$ is the d×d identity matrix, which indicates that the noise between each action dimension is independent of each other, ensuring that each dimension of the action is perturbed independently. The function $\mathrm{clip}(\cdot ,\;-a_{\max},\;a_{\max})$ restricts each element of the noisy action to remain within the valid action range $\left[-a_{\max},\;a_{\max}\right]$, where $a_{\max}$ is the maximum action magnitude allowed by the environment. In order to evaluate the control performance of each algorithm in an anti-interference environment, this section conducts a comparative analysis from two dimensions: trajectory tracking accuracy and speed error.

**Figure 6:**
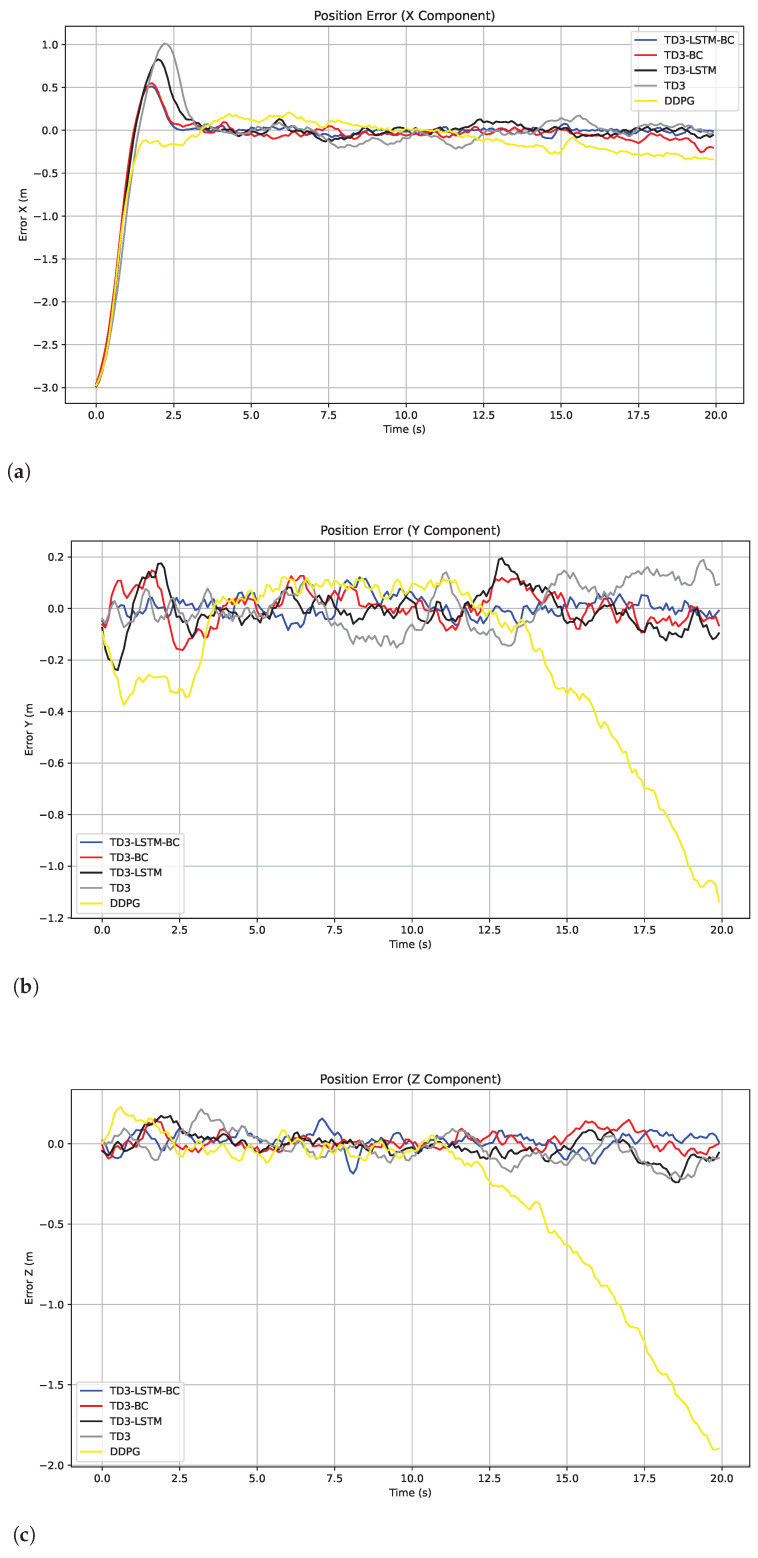
Tracking errors in the x, y, and z directions in Experiment 3. (**a**) Tracking errors in the x. (**b**) Tracking errors in the y. (**c**) Tracking errors in the z.

Figure 6 shows the error curves of the five algorithms after adding Gaussian perturbations. TD3-LSTM-BC achieves significantly lower RMSE and MAE under noise perturbations, and the three-axis error fluctuations are the most stable. DDPG has the most unstable error, especially the *y*-axis and *z*-axis errors gradually diverge with the disturbance, reflecting its weak anti-interference ability. Although TD3 has improved compared to DDPG, it still has large oscillations in the *y*-axis direction. Both TD3-LSTM and TD3-BC have shown significant improvements on the basis of TD3. The former has better transient response on the *x*-axis, and the latter has faster initial convergence, which is consistent with the training performance results. The MAE comparison of the five algorithms in the disturbance and non-disturbance environments is shown in Table 4, and the RMSE comparison is shown in Table 5. Among them, w/o Dist. means that the index is measured without disturbance, and w/ Dist. means that the index is measured after disturbance.

**Figure 7:**
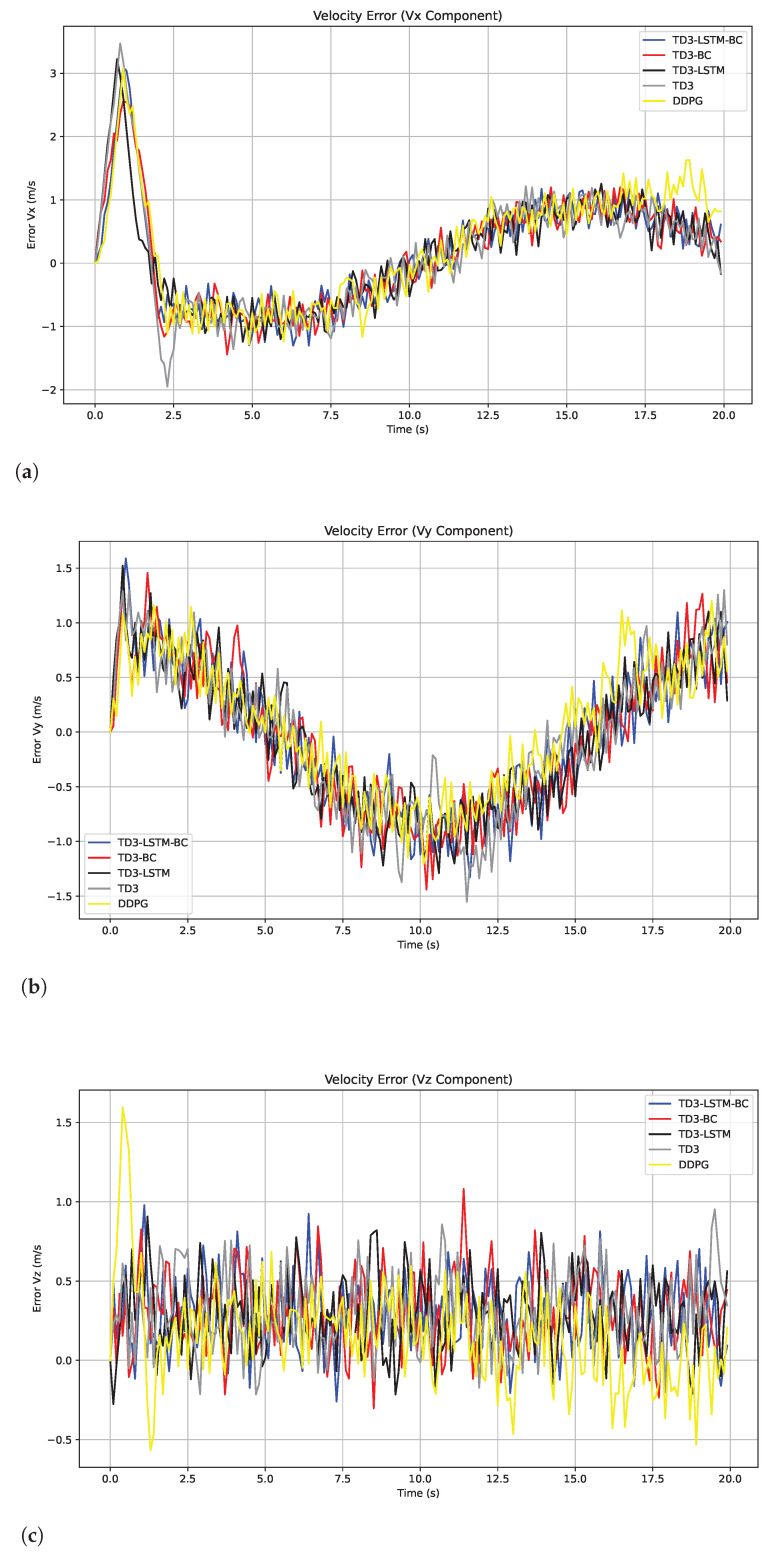
Tracking errors of velocity in x, y, and z directions in Experiment 3. (**a**) Tracking errors of velocity in x. (**b**) Tracking errors of velocity in y. (**c**) Tracking errors of velocity in z.

In terms of speed tracking, as shown in Figure 7, TD3-LSTM-BC still maintains its leading advantage, and the speed error in the dynamic maneuvering stage is significantly lower than that of other algorithms. Although DDPG has a small steady-state speed error, this is because its strategy is prone to fall into local optimality and adopts a “conservative slow-forward” control method, which leads to large accumulation of trajectory position errors due to slow response and limited action amplitude [36]. TD3 is more stable than DDPG, but there is still speed overshoot in the rapid change stage. Its deterministic strategy lacks memory of historical states, which limits its anti-interference ability. In contrast, TD3-LSTM-BC, which combines LSTM time series modeling and behavioral cloning strategy initialization, effectively alleviates the above problems and achieves smoother speed tracking under complex time-varying disturbances. The results show that although the local optimality of DDPG can present good speed error under constrained conditions, the integrated architecture of TD3-LSTM-BC shows stronger robustness and control accuracy over the entire trajectory range. The MAE comparison of the five algorithms in the disturbance and non-disturbance environments is shown in Table 6, and the RMSE comparison is shown in Table 7.

### 5.4. Generalization Performance

The experiments in this section tested the generalization ability of the reinforcement learning agent. In the three experiments described above, the interval between each step in the drone environment was 0.1 s, equivalent to a 10Hz update frequency. In this section, the interval was 0.05 s, equivalent to a 20Hz update frequency.

**Figure 8:**
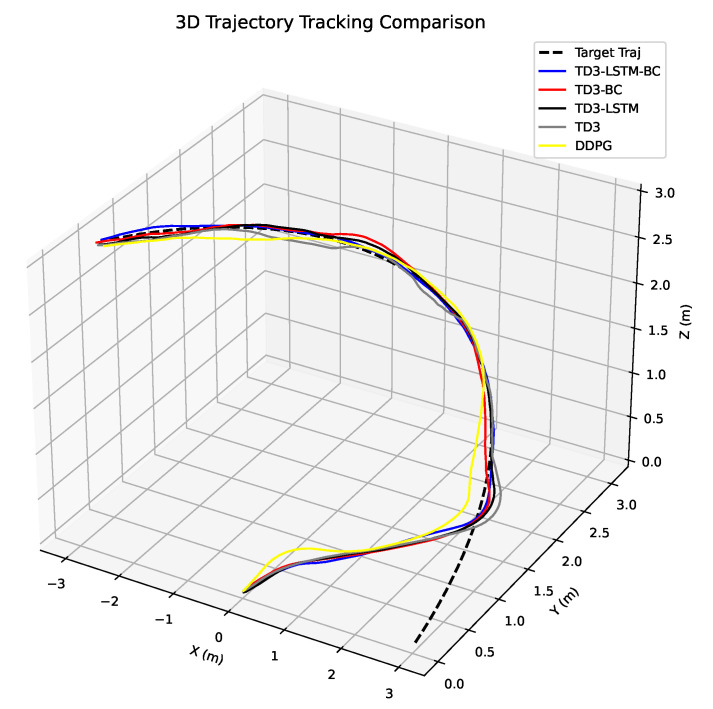
3D display of UAV tracking trajectory in Experiment 4.

**Figure 9:**
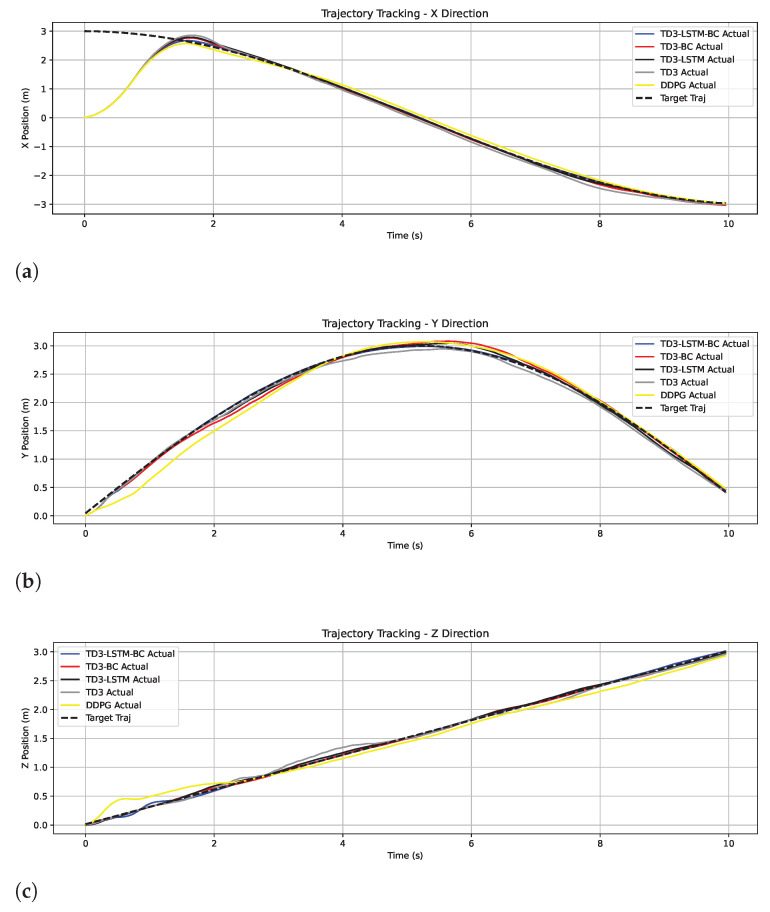
Tracking trajectories in the x, y, and z directions in Experiment 4. (**a**) Tracking trajectories in the x. (**b**) Tracking trajectories in the y. (**c**) Tracking trajectories in the z.

**Figure 10:**
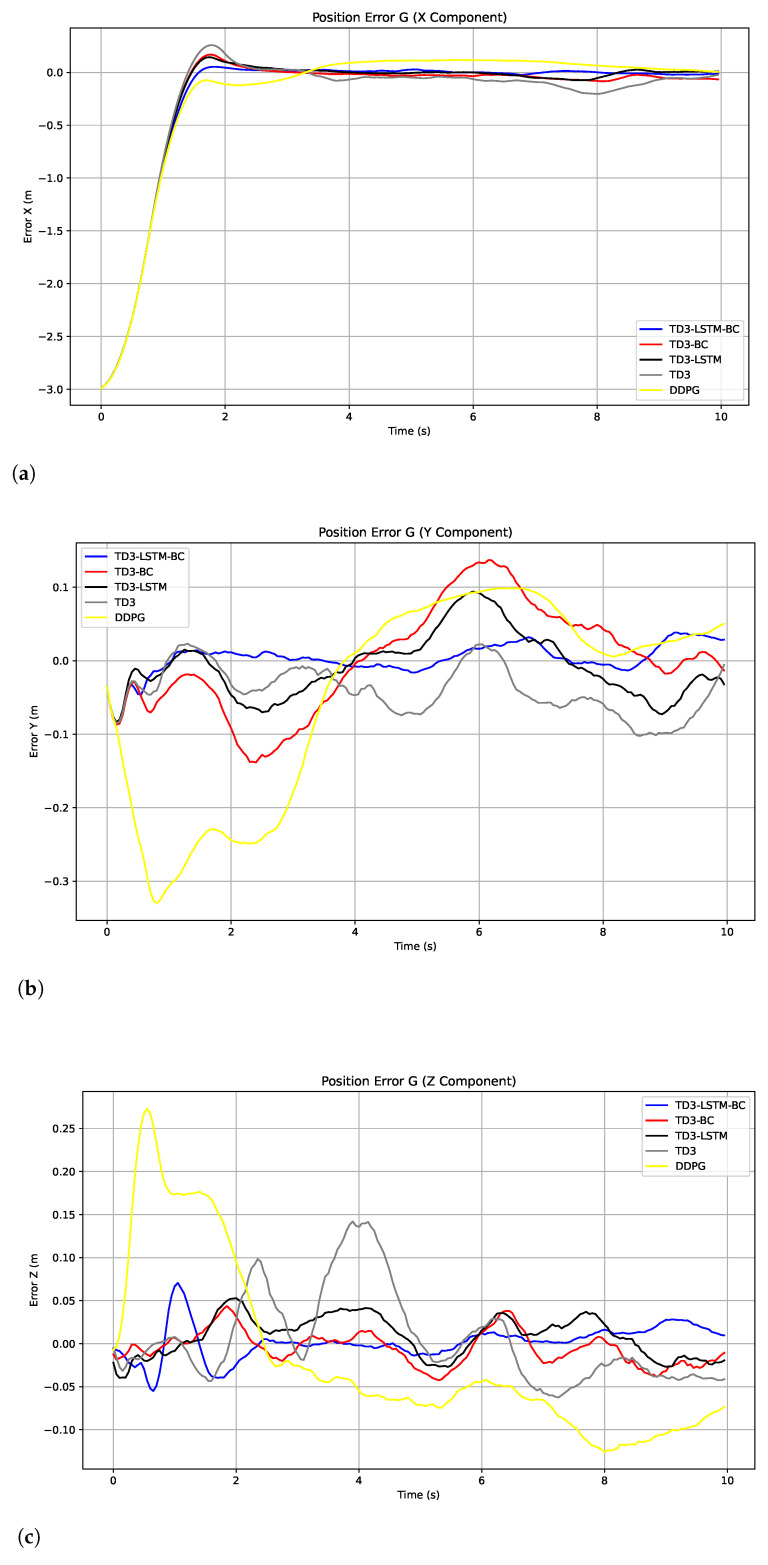
Tracking errors in the x, y, and z directions in Experiment 2. (**a**) Tracking errors in the x. (**b**) Tracking errors in the y. (**c**) Tracking errors in the z.

**Figure 11:**
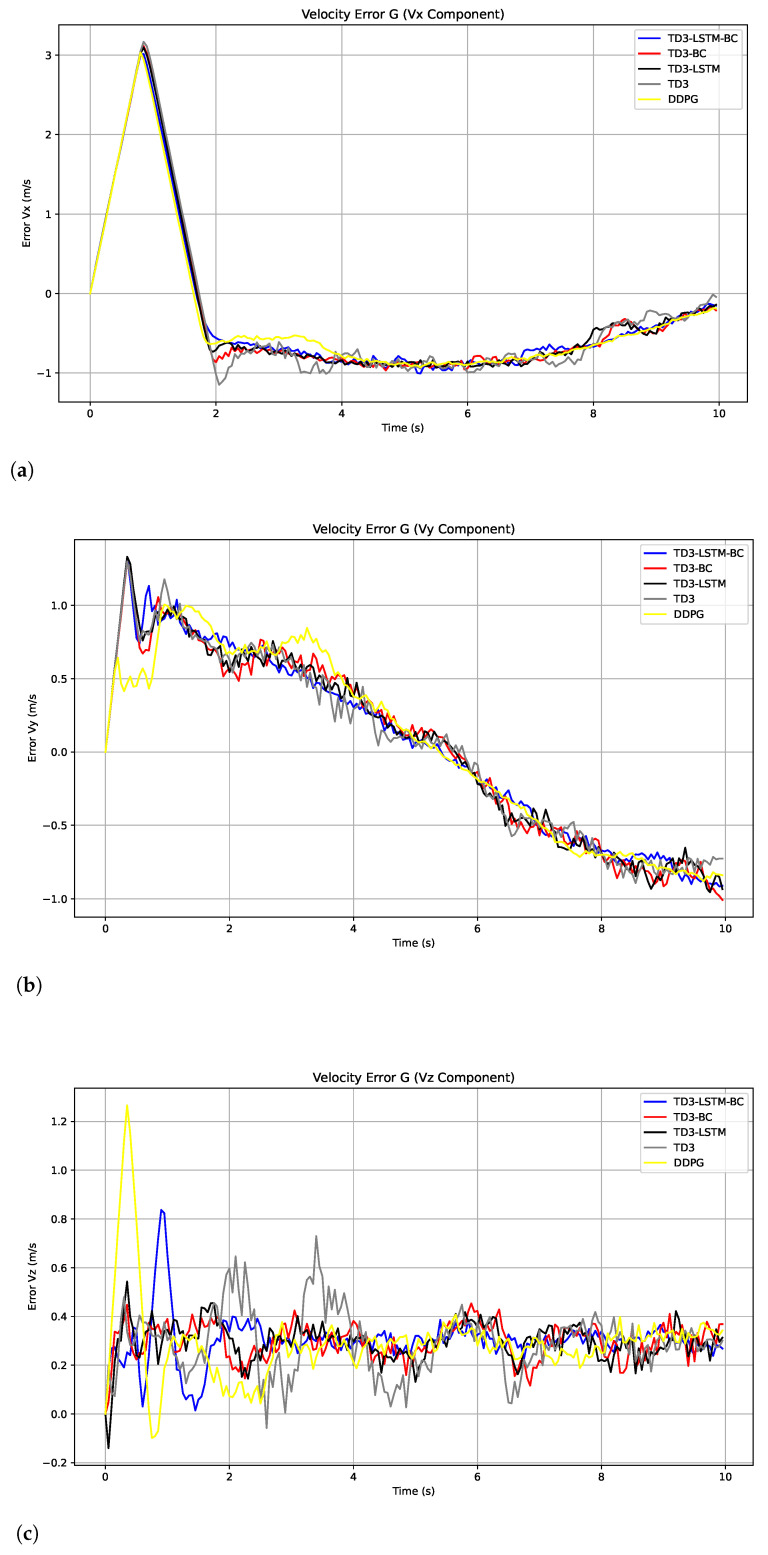
Tracking error of velocity in x, y, and z directions in Experiment 2. (**a**) Tracking error of velocity in x. (**b**) Tracking error of velocity in y. (**c**) Tracking error of velocity in z.

The experimental results are shown in Figure 8, Figure 9, Figure 10 and Figure 11. Figure 8 illustrates the trajectory tracking performance of different algorithms in three-dimensional space. It can be seen that TD3-LSTM-BC tracks the reference trajectory most stably and closely, with almost no noticeable deviation in turns and complex trajectory sections. TD3-LSTM and TD3-BC perform second best, remaining roughly close to the target trajectory but exhibiting some lag in areas of rapid change. DDPG and TD3, on the other hand, tend to deviate significantly during dynamic responses, resulting in relatively poor tracking stability.

Figure 9 shows a comparison of tracking trajectories in the *x*, *y*, and *z* directions. The TD3-LSTM-BC curve almost completely overlaps with the target curve, demonstrating strong tracking performance. TD3-LSTM and TD3-BC also track well but exhibit slight phase differences in some sections. In contrast, the DDPG and TD3 curves exhibit significant deviation and significant oscillation.

Figure 10 shows the position tracking errors of each algorithm along the three axes. TD3-LSTM-BC achieves the smallest error amplitude and the narrowest fluctuation range, demonstrating its ability to maintain good accuracy and stability even under high-frequency control. The error curves of TD3-LSTM and TD3-BC are slightly higher but remain within a relatively small range overall. However, the errors of DDPG and TD3 are larger, particularly with significant error peaks during rapid trajectory changes.

Figure 11 illustrates the evolution of velocity error. The results show that TD3-LSTM-BC maintains the smallest fluctuation and the smoothest response curve in velocity tracking. The velocity errors of TD3-LSTM and TD3-BC are relatively small but still exhibit some jitter, while the velocity error curves of DDPG and TD3 exhibit more pronounced oscillations. Overall, TD3-LSTM-BC performed best in this experiment because it combines the temporal modeling capabilities of LSTM with the prior knowledge guidance of behavior cloning. This allows it to capture the temporal dependencies in trajectory control while leveraging expert experience to stabilize the training process. As a result, it significantly outperforms other algorithms in both position accuracy and velocity stability, demonstrating the strongest generalization ability.

## 6. Conclusions

Target tracking control for UAVs in complex dynamic environments remains a critical challenge due to the limitations of conventional RL algorithms in partially observable and time-varying scenarios. To address this issue, this paper proposes a biomimetically inspired TD3-LSTM-BC framework, which integrates an LSTM network for temporal state modeling and BC for policy initialization. The LSTM module mimics the memory and temporal reasoning capabilities of biological neural systems, enabling the agent to infer system dynamics from sequential observations. Meanwhile, the BC module draws on expert demonstrations to guide early-stage learning, resembling imitation learning in natural organisms and significantly enhancing training efficiency and stability. Simulation results demonstrate that the proposed TD3-LSTM-BC method outperforms baseline algorithms in terms of learning performance and robustness, highlighting its potential as a bio-inspired control solution for autonomous UAVs operating in dynamic and uncertain environments.

Nevertheless, this study has several limitations. First, the proposed method has only been validated in simulation, and deployment verification on actual UAV hardware remains to be explored. Second, the current work focuses on single-UAV trajectory tracking, while performance in multi-UAV collaborative scenarios is yet to be investigated. Third, the computational complexity of the algorithm may affect real-time applicability, which calls for further optimization. These aspects will be the focus of future research to advance the practical deployment of the proposed approach.

## Figures and Tables

**Table 1:** UAV simulation parameters (Newton–Euler model).

Category	Parameter	Value
Physical Properties	Mass (*m*)	1.0 kg
	Linear Acceleration Bound	± 5.0 m/$s^{2}$
	Angular Acceleration Bound	± 5.0 rad/$s^{2}$
	Euler Angle Constraints	Roll/Pitch: [−π/2,π/2]
		Yaw: [−π,π]
State Space	Position (x,y,z)	$R^{3}$ (unbounded)
	Velocity (vx,vy,vz)	$R^{3}$
	Attitude (roll,pitch,yaw)	[−π/2,π/2]×[−π/2,π/2]×[−π,π]
	Angular Velocity	$R^{3}$
Action Space	Linear + Angular Acc.	6D: ${\left[-5.0,5.0\right]}^{6}$
Simulation	Time Step (Δt)	0.1 s
	Max Episode Steps	200

**Table 2:** MAE and RMSE of tracking position errors of five algorithms.

	Algorithms	TD3-LSTM-BC	TD3-BC	TD3-LSTM	TD3	DDPG
Axis						
X	MAE	0.1249	0.1415	0.1394	0.1877	0.5088
	RMSE	0.5033	0.5057	0.5039	0.5115	0.7492
Y	MAE	0.0113	0.0526	0.0477	0.0771	0.6744
	RMSE	0.0157	0.0675	0.0600	0.0894	1.0208
Z	MAE	0.0148	0.0327	0.0461	0.0630	0.7880
	RMSE	0.0183	0.0471	0.0690	0.0800	1.1669
Overall	MAE	0.0503	0.0756	0.0777	0.1093	0.6571
	RMSE	0.1791	0.2068	0.2110	0.2270	0.9789

**Table 3:** MAE and RMSE of tracking velocity errors of five algorithms.

	Algorithms	TD3-LSTM-BC	TD3-BC	TD3-LSTM	TD3	DDPG
Axis						
X	MAE	0.7169	0.7235	0.7158	0.7416	0.7370
	RMSE	0.8493	0.8570	0.8534	0.8902	0.8492
Y	MAE	0.5519	0.5578	0.5595	0.5477	0.5239
	RMSE	0.6261	0.6237	0.6275	0.6308	0.5885
Z	MAE	0.2962	0.2929	0.2936	0.3049	0.2031
	RMSE	0.3124	0.3166	0.3130	0.3418	0.2897
Overall	MAE	0.5216	0.5247	0.5230	0.5314	0.4880
	RMSE	0.5959	0.5991	0.5980	0.6210	0.5758

**Table 4:** Comparison of the MAE of position error with and without interference.

	Algorithms	TD3-LSTM-BC	TD3-BC	TD3-LSTM	TD3	DDPG
Axis						
X	w/o Dist.	0.1249	0.1415	0.1394	0.1877	0.5088
	w/ Dist.	0.1291	0.1512	0.1701	0.2240	0.5009
Y	w/o Dist.	0.0113	0.0526	0.0477	0.0771	0.6744
	w/ Dist.	0.0125	0.0587	0.0486	0.0861	0.6519
Z	w/o Dist.	0.0148	0.0327	0.0461	0.0630	0.7880
	w/ Dist.	0.0162	0.0468	0.0494	0.0769	0.7821
Overall	w/o Dist.	0.0503	0.0756	0.0777	0.1093	0.6571
	w/ Dist.	0.0526	0.0856	0.0894	0.1290	0.6450

**Table 5:** Comparison of the RMSE of position error with and without interference.

	Algorithms	TD3-LSTM-BC	TD3-BC	TD3-LSTM	TD3	DDPG
Axis						
X	w/o Dist.	0.5033	0.5057	0.5039	0.5115	0.7492
	w/ Dist.	0.5051	0.5208	0.5321	0.5559	0.7591
Y	w/o Dist.	0.0157	0.0675	0.0600	0.0894	1.0208
	w/ Dist.	0.0172	0.0750	0.0586	0.0963	0.9653
Z	w/o Dist.	0.0183	0.0471	0.0690	0.0800	1.1669
	w/ Dist.	0.0198	0.0578	0.0666	0.0931	1.1552
Overall	w/o Dist.	0.1791	0.2068	0.2110	0.2270	0.9789
	w/ Dist.	0.1807	0.2179	0.2191	0.2484	0.9599

**Table 6:** Comparison of the MAE of velocity error with and without interference.

	Algorithms	TD3-LSTM-BC	TD3-BC	TD3-LSTM	TD3	DDPG
Axis						
X	w/o Dist.	0.7169	0.7235	0.7158	0.7416	0.7370
	w/ Dist.	0.7205	0.7340	0.7668	0.7856	0.7795
Y	w/o Dist.	0.5519	0.5578	0.5595	0.5477	0.5239
	w/ Dist.	0.5532	0.5653	0.5671	0.5680	0.5186
Z	w/o Dist.	0.2962	0.2929	0.2936	0.3049	0.2031
	w/ Dist.	0.2981	0.3094	0.3225	0.3217	0.2593
Overall	w/o Dist.	0.5216	0.5247	0.5230	0.5314	0.4880
	w/ Dist.	0.5239	0.5362	0.5521	0.5584	0.5192

**Table 7:** Comparison of the RMSE of velocity error with and without interference.

	Algorithms	TD3-LSTM-BC	TD3-BC	TD3-LSTM	TD3	DDPG
Axis						
X	w/o Dist.	0.8493	0.8570	0.8534	0.8902	0.8492
	w/ Dist.	0.8510	0.8764	0.9438	0.9376	0.8951
Y	w/o Dist.	0.6261	0.6237	0.6275	0.6308	0.5885
	w/ Dist.	0.6275	0.6536	0.6540	0.6619	0.6032
Z	w/o Dist.	0.3124	0.3166	0.3130	0.3418	0.2897
	w/ Dist.	0.3142	0.3647	0.3922	0.3814	0.3568
Overall	w/o Dist.	0.5959	0.5991	0.5980	0.6210	0.5758
	w/ Dist.	0.5976	0.6316	0.6633	0.6603	0.6184

## Data Availability

The original contributions presented in this study are included in the article material. Further inquiries can be directed to the corresponding author.
